# Odontogenic Subperiosteal Abscess of the Lateral Orbit: Timely Recognition and Management

**DOI:** 10.1055/s-0041-1731588

**Published:** 2021-08-13

**Authors:** Ashley N. Houle, Chau Pham, Nita Valikodath, Jordan S. Elmowitz, Nicholas Callahan

**Affiliations:** 1 Chicago Medical Center, The University of Illinois, Chicago, Illinois, United States

**Keywords:** abscess, orbit, pediatric, subperiosteal

## Abstract

Orbital abscess is a rare entity due to an odontogenic infection. The progression from a toothache to serious complications such as blindness or death can be sudden and severe. The authors present the case of a 13-year-old male patient with a 2-day history of dental pain, which progressed to involve the periorbital tissues. He was experiencing visual symptoms. Computed tomographic imaging revealed a canine space abscess associated with a carious right maxillary molar in continuity with a subperiosteal abscess of the right lateral orbit. Surgical drainage was performed under general anesthesia via intraoral and extraoral approaches. The postoperative course was uncomplicated and vision improved. Multidisciplinary and timely management is crucial for successful outcomes in managing orbital abscesses of odontogenic origin. Therefore, it is crucial for emergency and primary care physicians to recognize when specialist consultation is indicated and expedite this process.

## Introduction

Orbital abscess secondary to an odontogenic cause is a rare, but dangerous complication that can result in permanent vision loss. Timely recognition and management are paramount. If untreated, this can lead to cavernous sinus thrombosis and/or a brain abscess resulting in death. 1 This is the case of a 13-year-old male with a 2-day history of dental pain which rapidly progressed to an abscess involving the periorbital tissues. Specialists were consulted in a timely fashion by the pediatric emergency physician. A multidisciplinary approach involving ophthalmology and oral and maxillofacial surgery specialists was necessary to manage this infectious process surgically. The presentation, extent of involvement, and surgical intervention are discussed in this case report.

## Patient Presentation

A 13-year-old previously healthy male was brought to the emergency department for a 2-day history of persistent right maxillary dental pain with associated swelling. He had recently received a routine dental evaluation and cleaning, and was planned for excavation and restoration of the dental caries within the month. One dose of 900 mg of intravenous clindamycin was administered at the outside hospital, and preseptal cellulitis was identified on the computed tomography (CT) scan per the outside hospital radiology written interpretation. The patient was transferred the same day to the University of Illinois Medical Center for specialty evaluation. At that time, the patient could not open his right eye due to swelling and reported blurry vision when manually opening his eyelids. He endorsed right-sided facial pain and pain with eye movement. He also presented with a 1-day history of subjective fevers and a recorded fever at the outside hospital.

**Fig. 1:**
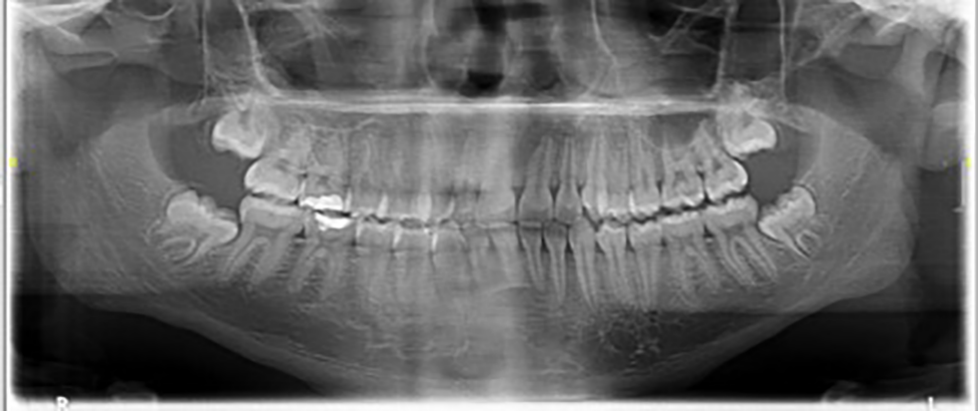
Panoramic radiograph demonstrating large restoration of tooth 3 with periapical radiolucency and right maxillary sinusitis.

Clinical examination revealed severe right periorbital and midface edema. Mild emphysema of the right upper eyelid was also noted. Ophthalmologic exam of the affected side revealed 20/20 vision without evidence of optic nerve compromise, mild restriction to adduction, and mildly elevated intraocular pressure. Intraorally, tooth 3 had a large restoration with adjacent fluctuant vestibular swelling ( Fig. 1 ). No active drainage was noted. The patient had a leukocytosis of 17.9, a neutrophilia of 84.4%, and an absolute neutrophil count of 15.1. The patient was admitted and started on 3,000 mg of intravenous ampicillin-sulbactam every 6 hours and 30 mg of intravenous ketorolac every 6 hours as needed for pain. Other vital signs and laboratory values were within normal limits.

**Fig. 2:**
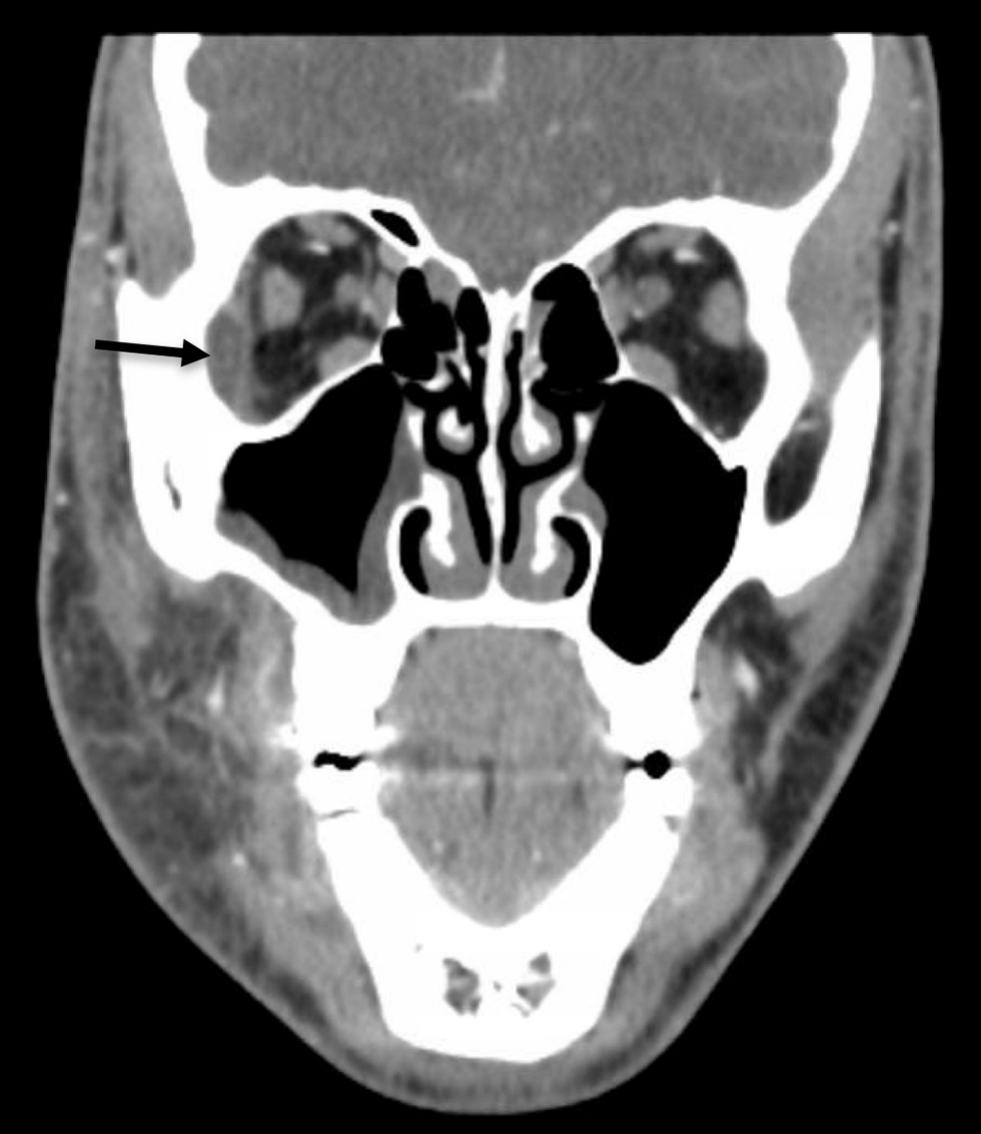
Computed tomography scan coronal section displayed the right lateral orbit subperiosteal abscess (black arrow) and right maxillary sinusitis.

Review of the outside hospital CT scan by the ophthalmology and oral and maxillofacial surgery services at our institution lead to the identification of a subperiosteal abscess at the right lateral orbit ( Fig. 2 ). The right canine space abscess emanated from the buccal roots of tooth 3. Continuity of the fluid collection was noted extending from the right canine space superiorly and posteriorly to the right lateral orbit. Right maxillary sinusitis was also apparent. A panoramic radiograph revealed previously restored tooth 3 with a periapical radiolucency. Intravenous antibiotics were continued and the patient was taken to the operating room in a timely fashion for surgical drainage.

**Fig. 3:**
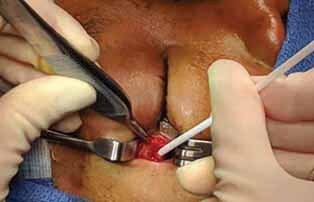
Intraoperative photo showing the culture procurement via the lateral canthotomy.

The patient underwent surgical drainage of the involved abscesses, as well as extraction of the associated molar, tooth 3. The anesthesia service provided general anesthesia via an oral endotracheal tube. The ophthalmology service provided local anesthesia via infiltration into right eye lids and right lateral canthal area using 1% lidocaine with 1:100,000 epinephrine. Next, the service performed an intraorbital incision and drainage through dissection of the preperiosteal plane along the lateral orbital rim, opening the periosteum and dissecting along the subperiosteal plane into orbit ( Fig. 3 ). The fluid and tissue were sent for culture. After copious irrigation and hemostasis, the tissues were closed primarily. The oral and maxillofacial surgery service provided local anesthesia via intraoral maxillary infiltration by administering 4 mL of 2% lidocaine with 1:100,00 epinephrine. Next, the service performed an intraoral incision and drainage of the right canine space abscess and extraction of tooth 3 using elevators and forceps. A quarter-inch Penrose drain was placed into the canine space after copious irrigation.

The patient’s postoperative course was uncomplicated. Cultures were positive for *Peptostreptococcus* micros and *Prevotella denticola* from the right orbital tissue and *Haemophilus parainfluenzae* , *Rothia mucilaginosa* , and *Streptococcus mitis* / *oralis* from the right canine space. As an inpatient, the patient’s antibiotic regimen consisted of 3,000 mg of intravenous ampicillin-sulbactam every 6 hours, 15 mL of 0.12% chlorhexidine gluconate solution swish, and spit two times per day, and application of 0.5% erythromycin ophthalmic ointment to the right eyelid four times per day. For pain control, the patient continued utilizing 30 mg of intravenous ketorolac every 6 hours, and for inflammation, the patient was given a single dose of 8 mg intravenous dexamethasone at 9:00 on postoperative day 1. The patient was discharged the following day with an antibiotic regimen consisting of oral 875 to 125 mg amoxicillin-clavulanic acid taken two times per day for 5 days, 0.12% chlorhexidine gluconate solution swish and spit two times per day for 7 days, and 0.5% erythromycin ophthalmic ointment applied to the right eyelid four times per day for 7 days. For pain control, the patient was prescribed 600 mg of oral ibuprofen taken every 6 hours for 7 days, and for inflammation, the patient was prescribed 4 mg of Medrol dose pack taken as directed for 6 days. The Penrose drain was removed on postoperative day 3, and the patient had notable improvements at his follow-up appointments as an outpatient.

## Discussion

Orbital abscess is an exceedingly rare complication arising from an odontogenic infection, with reports of <5% incidence. 2 Orbital inflammation can quickly lead to permanent disability or even death. 3 The classification of periorbital and orbital inflammation is grouped into the following categories: (1) inflammatory edema/preseptal cellulitis, (2) orbital cellulitis, (3) subperiosteal abscess, (4) orbital abscess, and (5) cavernous sinus thrombosis. A subperiosteal abscess results from purulent material collecting between the periorbital and the orbital bones. 4 This may lead to orbital pressures quickly rising and cause visual impairment and further progression of the infection. 5 Clinical signs of orbital involvement include limitation in extraocular motility, symptomatic binocular diplopia, firmness to retropulsion, and evidence of relative afferent pupillary defect.

Odontogenic infections follow the path of least resistance. The most common etiology of orbital abscesses in the pediatric population is maxillary or ethmoidal sinusitis. 2 Infections of the maxillary teeth commonly involve the sinuses. 6 Odontogenic source only accounts for 1.3 to 2% of orbital infections, so this is rarely seen. 7 In this case, the subperiosteal abscess resulted from the odontogenic infection caused by the gross decay of tooth 3 traveling upward through the canine space and maxillary sinus, and posteriorly to the subperiosteal region of the lateral orbit. Direct extension of bacteria into the subperiosteal space is made possible by neurovascular foramina, congenital and acquired bony dehiscence, and the valveless venous anastomoses. 8 Subperiosteal abscesses are typically polymicrobial with reports of both anaerobic and aerobic bacteria. 9 The oral flora is usually the originating source. 10 Rapid expansion of the subperiosteal abscess can occur and this sits in a relatively avascular zone, making it difficult for antibiotics to penetrate and suppress bacterial growth. 8

Patients with visual symptoms or severe periorbital edema with evidence of restriction in extraocular motility should have immediate assessment by a specialist, and CT imaging is indicated. Treatment of an orbital abscess with emergent drainage is suggested any time patients report visual disturbance, and even those without. 11 Surgical intervention facilitates normalization of the orbital pressure and also establishes aerobic conditions. 5 The Garcia-Harris criteria is used to help decide whether surgical intervention is recommended. This is based on the age of the patient at presentation and the location of the infection. 8 For a patient presenting with a subperiosteal orbital abscess, nonsurgical treatment with close observation and intravenous antibiotics is recommended only if the patient is less than 9 years of age, there is no intracranial involvement, no frontal sinus involvement, no dental abscess, no vision loss or afferent pupillary defect, and the medial wall abscess is of moderate or smaller size. 12 Close follow-up and administration of antimicrobial therapy is essential. 13 Surgery becomes indicated if the aforementioned criteria is not met, if the infection involves the optic nerve, or if there is suspicion for an infection with anaerobic bacteria. 12

## Conclusion

Orbital abscess secondary to an odontogenic cause is a rare, but dangerous complication that can result in permanent vision loss. Dental pain is a common complaint among pediatric patients presenting to emergency departments, dental offices, and primary care providers. Periorbital edema and visual changes should alert the provider to escalate care. Timely recognition and management are paramount. In the present case, the patient had timely intervention resulting in resolution of symptoms and return to function.
